# Real-World Attainment of Low-Density Lipoprotein Cholesterol Goals in Patients at High Risk of Cardiovascular Disease Treated with High-Intensity Statins: The TERESA Study

**DOI:** 10.3390/jcm12093187

**Published:** 2023-04-28

**Authors:** Vivencio Barrios, Xavier Pintó, Carlos Escobar, Jose F. Varona, José M. Gámez

**Affiliations:** 1 Department of Cardiology, University Hospital Ramón y Cajal, Alcalá University, 28034 Madrid, Spain; 2 Lipid and Vascular Risk Unit, Department of Internal Medicine, University Hospital of Bellvitge-Idibell-UB-CiberObn, 08907 L’Hospitalet de Llobregat, Spain; xpinto@bellvitgehospital.cat; 3 Department of Cardiology, University Hospital La Paz, 28046 Madrid, Spain; escobar_cervantes_carlos@hotmail.com; 4 Department of Internal Medicine, University Hospital HM Monteprincipe, HM Hospitales, 28660 Madrid, Spain; jfvarona@hmhospitales.com; 5 Department of Cardiology, University Hospital Son Llàtzer, 07198 Palma, Spain; jmgamez3@gmail.com; 6 CIBER de Fisiopatología de la Obesidad y la Nutrición (CIBEROBN CB 12/03/30038), Instituto de Salud Carlos III, 28029 Madrid, Spain

**Keywords:** cardiovascular diseases, atorvastatin, rosuvastatin, ezetimibe, low-density lipoprotein cholesterol, cardiovascular prevention, Spain

## Abstract

Despite steady improvements in cardiovascular disease (CVD) prevention, a scarce proportion of patients achieve the recommended LDL-C goals, even under high-intensity lipid-lowering therapy (LLT). Our study aimed to evaluate the attainment rate of LDL-C targets recommended by the 2019 European guidelines, and to characterize potential factors associated with LDL-C goal achievement and change patterns in LLT. We conducted a retrospective, observational study on patients treated with high-intensity atorvastatin or rosuvastatin ± ezetimibe at cardiology and internal medicine clinics across Spain. It included 1570 evaluable patients (median age: 62 years; established CVD: 77.5% [myocardial infarction: 34.3%]; and 85.8% at very high cardiovascular risk). Rosuvastatin ± ezetimibe was the LLT in 52.2% of patients, and atorvastatin ± ezetimibe in 47.8%. LLT had been modified in 36.8% of patients (side effects: 10%), being the most common switch from atorvastatin- to rosuvastatin-based treatment (77.2%). The risk-based LDL-C goal attainment rate was 31.1%, with 78.2% high-risk and 71.7% very high-risk patients not achieving the recommended LDL-C targets. Established CVD and familial hypercholesterolemia were significantly associated with the non-achievement of LDL-C goals. Although having limitations, this study shows that the guideline-recommended LDL-C goal attainment rate is still suboptimal despite using high-intensity statin therapy in a real-world setting in Spain.

## 1. Introduction

Cardiovascular disease (CVD) is the leading cause of death globally [1]. Elevated low-density lipoprotein cholesterol (LDL-C) is a major risk factor for CVD, given the causal role of LDL-C in the pathogenesis of atherosclerosis [2]. Lowering LDL-C levels is associated with a decreased risk of major cardiovascular (CV) events [3]. Based on this evidence, the European Society of Cardiology/European Atherosclerosis Society (ESC/EAS) guidelines recommend lowering LDL-C to an established target of LDL-C of <70 mg/dL and <55 mg/dL, in addition to ≥50% LDL-C reduction from baseline levels, for patients at high and very high CV risk, respectively [4]. Lowering LDL-C levels to guideline-recommended goals is the mainstay of CVD prevention. Intensive lipid-lowering therapy (LLT) with statins, especially high-intensity statins such as atorvastatin and rosuvastatin, efficiently reduces LDL-C and, consequently, decreases CVD risk [5], as recognized by current practice guidelines that recommend its use for CVD prevention in patients at high and very high risk [4]. Indeed, a direct correlation between high-intensity statins and LDL-C target achievement has been demonstrated [6]. Moreover, the combination of statins with ezetimibe has been shown to reduce the risk of CV events by lowering LDL-C [7].

During the last decades, primary and secondary prevention improvements have been associated with one-third of the decline in the cardiovascular mortality rate in developed countries [8,9]. Nevertheless, despite treatment guideline recommendations, low rates of LDL-C target achievement are reported, particularly in patients at very high CV risk. Thus, the EUROASPIRE V survey performed in 27 European countries revealed that only 29% of very-high risk patients reached the LDL-C target of <70 mg/dL. In the Spanish subset of the EUROASPIRE study, 49% of subjects achieved their LDL-C goals [10,11]. Similar rates were obtained in three Spanish studies, where around 55% of patients at very high CV risk achieved LDL-C <70 mg/dL [12,13,14]. Previous Spanish registries showed even lower rates of LDL-C target achievement. In the CODIMET, ENRICA, and REPAR studies, only 11.6%, 5.2%, and 26% of patients reached LDL-C levels <70 mg/dL, respectively [15,16]. Unfortunately, despite the advances in LDL-C-lowering treatment for LDL-C goal achievement during the last decades, the management of CVD prevention is still far from optimal and additional effort in LDL-C reduction should be pursued [17].

Considering that therapy with high-intensity statins (in monotherapy or in combination with ezetimibe) has shown an effective reduction in LDL-C levels, it is of utmost importance to re-evaluate and potentially reinforce the pharmacological management of patients who, even when being treated with high-intensity statin therapy, do not receive the maximally tolerated dose of statins for optimally reducing LDL-C levels or the recommended combination therapy with ezetimibe when LDL-C targets are not achieved, despite taking the maximally tolerated statin dose.

This study aimed to assess the attainment rate of risk-based LDL-C targets recommended by the 2019 ESC/EAS guidelines in primary and secondary prevention patients on high-intensity statin therapy with atorvastatin or rosuvastatin alone or combined with ezetimibe, in the real-world clinical setting in Spain. 

## 2. Materials and Methods

### 2.1. Study Design and Patients

The TERESA study (for its Spanish acronym of Consecución de Objetivos Terapéuticos En pacientes de alto Riesgo con Estatinas de alta potencia Solas o Asociadas a ezetimibe) was a multicenter, retrospective, observational study conducted at cardiology and internal medicine clinics in Spain. The study was conducted in accordance with the World Medical Association Declaration of Helsinki, all its amendments, and national regulations. The Independent Ethics Committee of Hospital Clínico San Carlos, Madrid (Spain), approved the study (SEC-EZE-2020-01), and written informed consent was obtained from all patients before their inclusion in the study. 

Consecutive adult patients (≥18 years) younger than 70 years who had been treated with high-intensity atorvastatin or rosuvastatin monotherapy or in combination with ezetimibe for primary or secondary prevention within the preceding three months before their inclusion in the study were eligible for inclusion. A retrospective chart review was conducted to collect data on the demographic and clinical characteristics of patients, laboratory data, LLT use and changes, and the attainment of risk-based LDL-C goals. 

### 2.2. Study Endpoints

The primary endpoint was the rate of risk-based LDL-C goal attainment according to the 2019 ESC/EAS guidelines [4]: <1.4 mmol/L (55 mg/dL) for very high risk, <1.8 mmol/L (70 mg/dL) for high risk, <2.6 mmol/L (100 mg/dL) for moderate-risk patients, and <116 mg/dL for low-risk patients. CV risk was assessed according to the 2019 ESC/EAS guidelines recommendations based on the presence of CVD, the coexistence of diabetes mellitus or chronic kidney disease, and other parameters to determine the risk through the Systematic Coronary Risk Evaluation (SCORE) chart proposed by the European guidelines at the time of the observation period [4]. LDL-C goal attainment was assessed according to the last available data on LDL-C levels in medical charts. For patients on LDL-C target, we additionally evaluated the achievement of ≥50% LDL-C reduction from baseline (defined as the first of the two last measurements recorded). Secondary endpoints included the evaluation of the factors associated with LDL-C goal attainment, including LLT used for LDL-C control and coexisting conditions, as well as the assessment of the changes in LLT, including those related to adverse events (AEs). 

### 2.3. Statistical Analysis

To describe quantitative variables, measures of central tendency and dispersion including the mean, standard deviation (SD), median, and interquartile range (IQR) were used. Counts and percentages were used to describe qualitative variables. For the comparison of categorical variables, the Chi-squared test was used. The Kolmogorov–Smirnov test and the Shapiro–Wilk test were used for checking the normality of data. 

Missing data were not considered in the analyses, and significance was considered at *p*-value < 0.05 for statistical testing. The statistical analyses were performed using the Statistical Package for the Social Sciences (SPSS) version 17.0 (SPSS Inc., Chicago, IL, USA).

## 3. Results

### 3.1. Patients

Between December 2020 and July 2021, a total of 1572 patients were enrolled in the study. Two patients were excluded due to non-compliance with eligibility criteria; therefore, 1570 subjects were finally evaluable for study analyses (Supplementary Figure S1). The demographic and clinical characteristics of the study population are shown in Table 1. Briefly, the median age was 62 (56–67) years, and 76.2% were male. Established CVD was documented in 77.5% of patients; specifically, myocardial infarction was recorded in 34.3%. Primary prevention patients (*n* = 384; 22.5%) had a median (IQR) SCORE of 4.5 (2.0–8.0). Overall, 85.8% of patients had a very high CV risk according to the 2019 ESC/EAS guidelines. 

### 3.2. Characterization of LLT Use and Changes 

Data on LLT at the time of the analysis showed that rosuvastatin monotherapy or in combination with ezetimibe was administered in 52.2% of patients and atorvastatin +/− ezetimibe in 47.8%. Rosuvastatin monotherapy was used in 11.1% of patients, most of whom (95.4%) received a dose of 20 mg. Rosuvastatin plus ezetimibe was used in 41% of patients, who mainly (88.7%) received rosuvastatin 20 mg plus ezetimibe 10 mg doses. Atorvastatin monotherapy was used in 26.8% of patients; among them, 56.2% were treated with a dose of 40 mg. The combination of atorvastatin and ezetimibe was administered in 21.1% of patients, with 58.9% being treated with atorvastatin 80 mg plus ezetimibe 10 mg. Combination therapy with ezetimibe, whether rosuvastatin or atorvastatin, was used in 62.1% of patients (Table 2). 

Lipid-lowering therapy had been changed in 578 (36.8%) patients. Prior LLT to that received by patients at the time of analysis consisted primarily of atorvastatin monotherapy (65.9%). Only 8.1% of patients had previously received atorvastatin plus ezetimibe combinations, while the proportion of patients receiving this combination increased significantly to 21.1% at the time of analysis (*p* < 0.001). Additionally, the use of atorvastatin at a daily dose of 80 mg in combination with ezetimibe had significantly increased from 1.9% to 12.4% (*p* < 0.001). Similarly, the proportion of patients on rosuvastatin plus ezetimibe combinations sharply increased from 3.9% to 41% (*p* < 0.001). The use of the dose of 40 mg of rosuvastatin in combination with ezetimibe was also higher, rising from 0.3% to 4.6% (*p* < 0.001) (Table 2). The LLT used by CV risk categories is shown in Table 2. Changes in LLT due to AEs were seen in 57 (9.9%) patients. Among these, the most common switch was from atorvastatin-based treatment to rosuvastatin-containing treatment (*n* = 44; 77.2%), with 30 (52.6%) patients changing from atorvastatin monotherapy to rosuvastatin plus ezetimibe due to undesirable side effects. Among those patients suffering AEs that modified their treatment without switching statins, 11 underwent changes involving dose adjustment or switched from monotherapy to combined therapy and vice versa (Supplementary Table S1). 

The presence of CVD was significantly associated with the type of LLT used (*p* < 0.001). Rosuvastatin and atorvastatin were used in a similar proportion of patients with an established diagnosis of CVD, while patients without a history of CVD were primarily treated with rosuvastatin. The diagnosis of familial hypercholesterolemia (FH) was also significantly associated with the type of LLT used (*p* < 0.001). Rosuvastatin alone or in combination was the LLT in 72.7% of subjects with FH, while atorvastatin-based therapy was the LLT in 27.3% (Supplementary Table S2).

### 3.3. Achievement of Risk-Based LDL-C Goals

**Figure 1:**
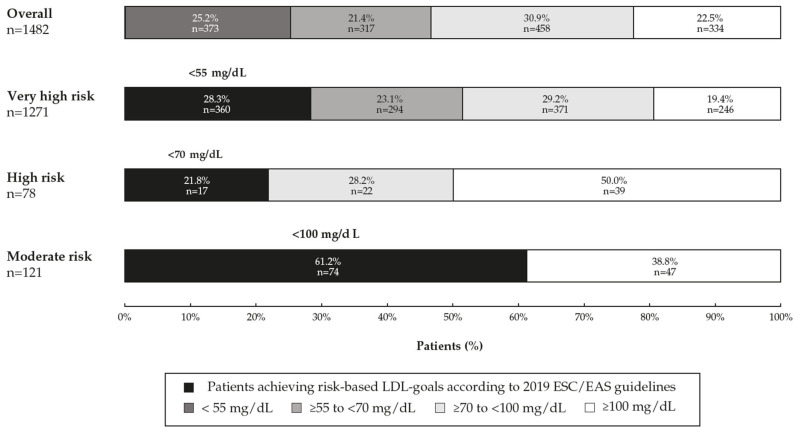
Distribution of LDL-C levels and attainment of LDL-C goals recommended by the 2019 ESC/EAS guidelines in the overall population and by patient cardiovascular risk (*n* = 1482). ESC/EAS: European Society of Cardiology/European Atherosclerosis Society.

Globally, 31.1% of the patients achieved the risk-based LDL-C goals according to the 2019 ESC/EAS guidelines. Notably, 78.2% of patients at high CV risk and 71.7% of very high-risk patients did not achieve the recommended LDL-C targets. Nearly half of the patients at very high risk (48.5%) had LDL-C levels ≥70 mg/dL (Figure 1). The median (IQR) LDL-C levels were 73 (55–98) and 83 (59–129.5) mg/dL in the last and preceding measurements, respectively. 

Among patients at very high CV risk in whom LDL-C goals were attained (*n* = 360), 33.5% (119/355) of patients had an LDL-C reduction ≥50%, respectively, while receiving high-intensity statin therapy. 

### 3.4. Factors Associated with LDL-C Goal Attainment 

**Figure 2:**
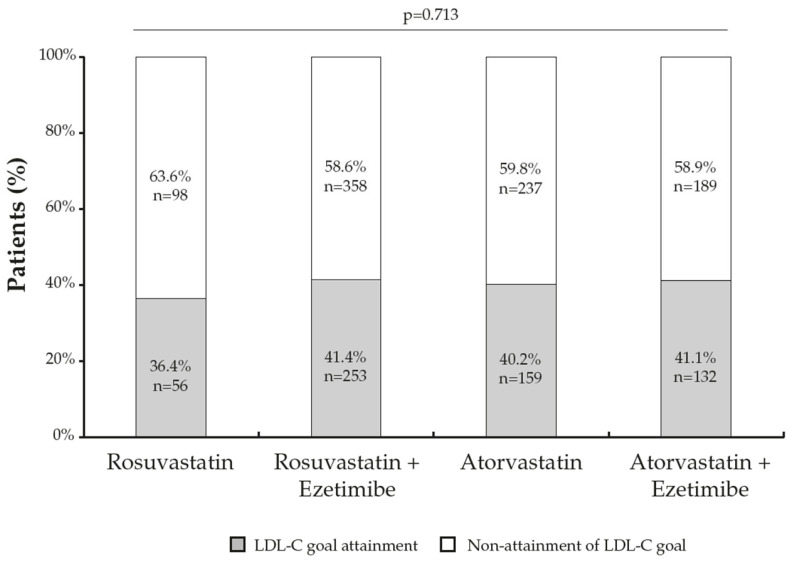
LDL-C goal attainment by lipid-lowering therapy (*n* = 1482).

**Figure 3:**
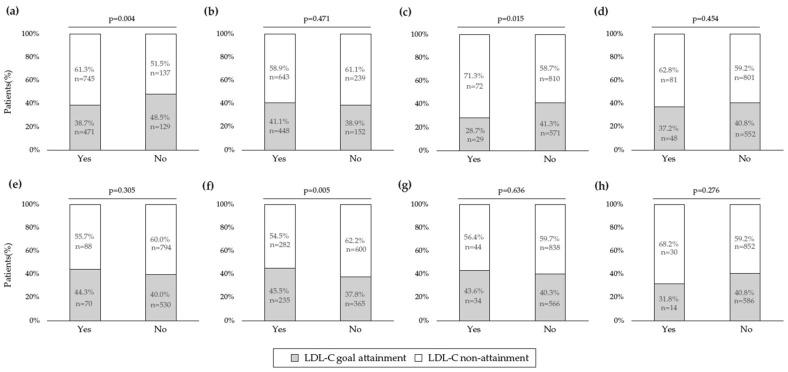
Factors associated with LDL-C goal attainment (*n* = 1482). (**a**) Cardiovascular disease; (**b**) Hypertension; (**c**) Familial hypercholesterolemia; (**d**) Chronic kidney disease; (**e**) Microalbuminuria; (**f**) Diabetes mellitus; (**g**) Retinopathy; (**h**) Neuropathy.

The risk-based LDL-C goal attainment did not differ according to the type of LLT (Figure 2). A statistically significant association was found between the presence of CVD and LDL-C goal achievement, with a lower proportion of patients with an established CVD reaching LDL-C targets than patients without a history of CVD (*p* < 0.001). Likewise, the diagnosis of FH was also significantly associated with the non-attainment of LDL-C goals, with a lower proportion of patients with FH who met LDL-C targets than patients without FH (*p* = 0.034). Diabetes mellitus was identified as a coexisting condition significantly associated with achieving LDL-C goals, with the non-achievement rate being higher among non-diabetic patients compared with diabetic patients (*p* = 0.010). The occurrence of chronic kidney disease or arterial hypertension was not significantly related to the achievement of the LDL-C goal (Figure 3).

## 4. Discussion

The TERESA study aimed to assess the achievement of guideline-recommended risk-based LDL-C goals in a nationwide cohort of patients on high-intensity statin therapy. This study shows suboptimal LDL-C goal achievement in primary or secondary prevention patients receiving high-intensity doses of atorvastatin or rosuvastatin (in monotherapy or combined with ezetimibe), with nearly 70% of very-high patients failing to achieve the recommended LDL-C goal, irrespective of LLT. This study also suggests that an established CVD and FH are associated with a lack of LDL-C goal achievement, according to the targets established in the 2019 ESC/EAS guidelines. Additionally, changes in LLT due to AEs mainly involve switching from atorvastatin- to rosuvastatin-based therapy.

We found an overall achievement rate for the risk-based LDL-C goals of only 31.1%, despite all patients being on high-intensity statins, even with 62% of them on combination therapy of high-intensity statins plus ezetimibe. Regardless of differences in the recommended LDL-C targets, baseline CV risk of patients, or LLT used for CVD prevention, our findings are consistent with several international and Spanish studies that have consistently reported low rates of LDL-C goal attainment in high- or very high-risk patients, ranging from 4% to 57% [6,10,11,12,13,14,17,18,19,20,21,22,23,24,25,26]. It must be highlighted that most patients included in the TERESA study were categorized as very high CVD risk as they were recruited in cardiology and internal medicine units. Overall, 91% of patients were at high or very high CV risk, and of these, only 21.8% and 28.3% of high-risk and very high-risk patients met the recommended risk-based LDL-C targets. These findings are in line with the European DA VINCI observational study, which showed that three-quarters of patients in primary and secondary care mainly on either moderate- or high-intensity statin monotherapy (53% and 32%, respectively) did not achieve the 2019 risk-based LDL-C goals [27]. Compared with the 2016 risk-based LDL-C goal attainment, which was already challenging, this study showed a lower proportion of patients who achieved their risk-based LDL-C goals recommended by the 2019 ESC/EAS guidelines, with more stringent criteria for LDL-C targets in very high-risk patients. Although high-intensity statins should reduce LDL-C by about 50% from baseline levels according to the 2019 dyslipidemia guidelines, less than 35% of very high-risk patients achieving LDL-C targets had a ≥50% reduction in LDL-C levels from baseline. These findings emphasize the utmost need for treatment revaluation and improvement in managing this group of patients of particular interest in achieving optimal LDL-C lowering, which may reduce CVD risk. Nevertheless, the low rate of achievement of the guideline-recommended LDL-C goals may also reflect a usual delay in the adoption and implementation of the 2019 guidelines into clinical practice, as the retrospective observation period covered the period from December 2020 to July 2021. Additionally, the COVID-19 pandemic may have affected lipid control mainly driven by delayed or limited access to health care due to restrictions, foregone outpatient visits, the shift from in-person visits to virtual care, and poor CVD risk factors management, including a reduction of routine laboratory monitoring [28,29], particularly during the first waves of the pandemic. 

The present study showed that established CVD was significantly associated with the non-attainment of LDL-C targets. Thus, a lower proportion of patients with a confirmed diagnosis of CVD achieved the recommended LDL-C goal compared with patients without CVD, as previously reported in the Spanish LYNX study [13]. On the contrary, other studies, including the international CEPHEUS study, showed that a history of CVD or previous acute myocardial infarction were associated with better LDL-C control rates [30,31,32]. Indeed, the presence of coronary artery disease (CAD) has been identified as having a highly significant and independent predictive role in the achievement of the recommended LDL-C goals [33,34], which may suggest more stringent monitoring and management of LLT to achieve the recommended therapeutic goals in patients with an established CVD. The worse LDL-C goal achievement in patients with CVD observed in our study might be explained by the more stringent 2019 LDL-C goals, the potential use of less-intensive therapies for lowering LDL-C levels, and the potential implications of the COVID-19 pandemic in secondary prevention, as previously reported [29]. 

Lowering elevated LDL-C levels is essential in FH patients, given the high risk of CV complications associated with FH [35]. Despite receiving guideline-recommended high-intensity statins, nearly 68% of FH patients did not attain their LDL-C goal in our series. The LDL-C goal attainment rate among FH patients observed in our study is comparable with that obtained in a UK study that included a similar population but was notably higher than previously shown in prior studies, such as the SAFEHEART study that reported a rate of LDL-C target attainment of only 10% in FH patients [36,37]. An LDL-C goal attainment rate widely ranging from 2% to 23% has been reported in European real-world studies [17]. The low LDL-C goal attainment rate among FH patients suggests that further clinical management efforts should be placed on these high-risk patients who may require more effective treatment options with new therapies and/or combination therapies, given that LDL-C target levels are frequently not reached with statins alone, likely due to the high baseline levels in these difficult-to-treat patients. 

In the current study, a higher proportion of patients with diabetes mellitus attained the recommended LDL-C goal compared with patients without diabetes, which is in agreement with many studies, including the Spanish LYNX and REPAR studies and the international CEPHEUS and DYSIS II studies, among others [13,15,25,26,31,38]. These findings suggest that clinicians may be paying more attention to CV risk factors management in diabetic patients in the real-world setting. However, still, nearly 54% of diabetic patients failed to achieve their risk-based LDL-C targets. 

A similar clinical benefit has been assumed for atorvastatin and rosuvastatin based on the benefit demonstrated for rosuvastatin in primary prevention patients, given the limited real-world studies and the lack of hard outcomes (morbidity and mortality) in clinical trials comparing these statins in secondary prevention. No differences have been reported between high doses of rosuvastatin and atorvastatin in CVD recurrence and support their use as clinically equivalent in secondary prevention [39]. We found that atorvastatin and rosuvastatin, either alone or in combination therapy, were used in a similar proportion of patients. Of note, rosuvastatin- and atorvastatin-based therapy was used similarly in patients with an established CVD or subjects in primary prevention. This observation may be related, at least in part, to the fact that the benefit may not depend on specific LLT, but on the effectiveness of the drug in reducing LDL-C [40]. However, rosuvastatin was mainly prescribed in primary prevention patients. 

The DA VINCI study highlighted the improvement in LDL-C lowering when more potent therapies for LDL lowering are used [27]. Shin J. et al. also recently suggested that high-intensity statins should be aggressively prescribed in patients with CVD to increase the LDL-C goal achievement rate [25]. Statins of high intensity for lowering LDL-C by at least 50% include rosuvastatin at doses of 20 and 40 mg/day and atorvastatin at doses of 40 and 80 mg/day. We found that about 56% of patients treated with atorvastatin monotherapy did not receive the higher dose of 80 mg, even though 85% of patients were at very high risk. Thus, many patients had yet to receive the maximally tolerated dose of statins for optimally reducing LDL-C levels. Of note, 5% of patients received the highest dose of rosuvastatin (40 mg), which was not marketed in Spain. The use of high-intensity statins in combination therapy has been associated with a higher likelihood of reaching LDL-C targets [7,26]. Accordingly, the 2019 ESC/EAS guidelines recommended the addition of ezetimibe to statin therapy in patients who are not at LDL-C targets, despite taking maximally tolerated statin doses. The present study showed that combination therapy with atorvastatin or rosuvastatin plus ezetimibe was used in about 60% of patients. However, there are a relatively high proportion of uncontrolled very high-risk patients on a maximally tolerated dose of statin therapy who may benefit from combination therapy but are not still receiving this approach in the real-world setting. 

While rosuvastatin-based therapy was the most frequent therapy for LDL-C lowering at the time of study analysis, atorvastatin monotherapy was the most common LLT previously received by patients in whom LLT had been changed. Interestingly, we found a significant increase in the proportion of patients treated with combinations of atorvastatin or rosuvastatin plus ezetimibe compared with prior treatment. Additionally, the use of the highest dose of atorvastatin and rosuvastatin within these combinations also showed a significant increase in comparison to the previous treatment. Although patients are far from achieving the recommended LDL-C treatment goals, the observed change in prescription patterns may suggest a prevention effort of clinicians to adhere to the 2019 guidelines regarding the optimization of LLT, particularly in very high-risk patients. 

In our study, 10% of modification in LLT was due to AEs. Adverse effects associated with statin therapy, notably muscle symptoms, can lead to non-compliance with the prescribed statin regimen. The incidence of AEs is therefore the major driver of statin switching [41]. A survey conducted in the United States (US) with more than 10,000 patients on statin therapy revealed that 28% of switches between statins were due to side effects [42]. In our cohort, the most frequent pattern of safety-related change in statin therapy was switching from atorvastatin ± ezetimibe to rosuvastatin ± ezetimibe (nearly 80% of them). This change may be related to a more favourable safety profile of rosuvastatin. Indeed, rosuvastatin has been reported to be better tolerated than atorvastatin [43,44,45,46]. However, available randomized controlled trials supporting the efficacy of high-intensity statin therapy in reducing CV events had not been powered to detect differences in AEs between high-intensity rosuvastatin and high-intensity atorvastatin. Additionally, rosuvastatin, which is not metabolized by cytochrome P450, has a lower risk of interactions [4]. A head-to-head comparison in adults with hypercholesterolemia conducted in the US and a meta-analysis of randomized trials with Asian populations showed a similar safety profile between rosuvastatin and atorvastatin [47,48]. However, a retrospective study conducted on more than 10,000 veterans on high-intensity statins in the US showed that atorvastatin was linked to an increased incidence of overall adverse drug reactions (ADRs), abnormal liver function, and muscle symptoms in subjects treated with atorvastatin compared with rosuvastatin [24]. Another small randomized study in patients with acute coronary syndrome found that rosuvastatin was significantly safer than atorvastatin regarding liver function [22]. Thus, further studies focused on safety would be warranted to better determine the potential differences in the safety profile of available high-intensity statins. Despite contradictory findings in the literature when comparing the safety profile of atorvastatin versus rosuvastatin, atorvastatin may be associated with an increased incidence of ADRs compared to rosuvastatin at the doses used, which could explain the changes in the treatment pattern observed in this study [22,24,49,50]. 

Some study limitations must be considered when interpreting the study findings. First, the retrospective nature of the study has conditioned data availability in medical charts, which were collected according to routine daily practice with no other purposes than clinical management. Additionally, local guidelines and the update of guideline-recommended LDL-C targets should be considered when comparing studies from different regions and periods. Furthermore, when interpreting LDL-C goal attainment and LLT used for CVD prevention, we must consider the usual delay in the implementation of clinical practice guidelines into practice. Additionally, the COVID-19 pandemic may have affected LDL-C control and LLT management. Despite the relevance of adherence to LLT for LDL-C goal attainment [51], data on therapeutic compliance with high-intensity statin therapy is unavailable in this study due to its retrospective nature. Lastly, the sample size was relatively small for patient subgroup analyses according to the CV risk factor, which limited the extraction of conclusions in the subjects at moderate or low risk, due to the representation of these patients being very scarce as patients were recruited in cardiology and the internal medicine units, where patients at high and very high CVD risk are typically managed. Nevertheless, this study provides valuable and updated real-world data on the attainment of LDL-C targets among high or very high-risk patients on high-intensity statin therapy in Spain.

## 5. Conclusions

This study has shown that the guideline-recommended LDL-C goal attainment rate is still suboptimal despite the use of high-intensity statin therapy in the real-world setting of Spain. Nearly 70% of very high-risk patients receiving atorvastatin or rosuvastatin in monotherapy or in combination with ezetimibe fail to achieve LDL-C targets. Nevertheless, the change in prescription patterns, with a significant increase in the use of rosuvastatin-based therapy, mainly combined with ezetimibe, to the detriment of atorvastatin use, together with the administration of higher statin doses, may suggest a prevention effort of clinicians to adhere to guideline recommendations on LLT optimization, particularly in very high-risk patients. Overall, most safety-related changes in LLT involve switching from atorvastatin- to rosuvastatin-based therapy. This study highlights the need to reconsider the management of patients who do not attain LDL-C goals even when treated with high-intensity LLT.

## Figures and Tables

**Table 1:** Patient demographic and clinical characteristics (*n* = 1570).

Patient Characteristics	Value
**Age**, median (IQR), years	62.0 (56.0–67.0)
**Sex**, male, *n* (%)	1196 (76.2)
**Body mass index**, median (IQR), kg/m^2^	28.1 (25.7–31.1)
**Comorbidity**	
At least one coexisting condition, *n* (%)	1539 (98.0)
Cardiovascular risk factors	
Arterial hypertension, *n* (%)	1148 (73.1)
Familial hypercholesterolemia, *n* (%)	121 (7.7)
Heterozygous familial hypercholesterolemia	111 (91.7)
Homozygous familial hypercholesterolemia	10 (8.3)
Chronic kidney disease, *n* (%)	132 (8.4)
Mean eGFR, mL/min/1.73 m^2^ (SD)	43.8 (12.8)
Microalbuminuria, *n* (%)	163 (10.4)
Diabetes mellitus, *n* (%)	545 (34.7)
Type 1, *n* (%)	23 (4.2)
Mean duration since diagnosis, years (SD)	22.5 (14.5)
Type 2, *n* (%)	522 (95.8)
Mean duration since diagnosis, years (SD)	9.1 (6.5)
Retinopathy, *n* (%)	79 (5)
Neuropathy, *n* (%)	45 (2.9)
**Current smoker**, *n* (%)	450 (28.7)
**Cardiovascular disease/events**, *n* (%)	1216 (77.5)
Coronary heart disease	686 (56.4)
Myocardium infarction	417 (34.3)
Stroke	42 (3.5)
Peripheral arterial disease	41 (3.4)
Cardiac valve disease	19 (1.6)
Others	11 (0.9)
**SCORE**, median (IQR) ^1^	4 (2–9)
**Cardiovascular risk (2019 ESC/EAS guidelines)**, *n* (%) ^2^	
Very high risk	1271 (85.8)
High risk	78 (5.3)
Moderate risk	121 (8.2)
Low risk	12 (0.8)

eGFR, estimated glomerular filtration rate; ESC/EAS, European Society of Cardiology/European Atherosclerosis Society; IQR, Interquartile range; SCORE: Systematic Coronary Risk Evaluation; SD, standard deviation. Valid % are used otherwise stated. ^1^ The SCORE was calculated over a population of 354 patients without previous cardiovascular events nor established cardiovascular disease (CVD); ^2^ overall, 88 patients without CVD and unavailability of data for SCORE calculation were excluded; percentages are calculated over 1482 patients.

**Table 2:** Lipid-lowering therapy.

Treatment	Last Treatment *n* = 1570 *n* (%)	Cardiovascular Risk			Prior Treatment *n* = 578 *n* (%)	*p*-Value Last vs. Prior Treatment ^1^
		Low-Moderate Risk *n* = 133 *n* (%)	High Risk *n* = 78 *n* (%)	Very High Risk *n* = 1271 *n* (%)		
**Rosuvastatin**, *n* (%)	175 (11.1)	27 (20.3)	10 (12.8)	117 (9.2)	127 (22.0)	<0.001
20 mg, *n* (%)	167 (10.6)	27 (20.3)	10 (12.8)	109 (8.6)	121 (20.9)	<0.001
40 mg, *n* (%)	8 (0.5)	0 (0.0)	0 (0.0)	8 (0.6)	6 (1.0)	0.177
**Rosuvastatin + Ezetimibe**, *n* (%)	644 (41.0)	47 (35.3)	35 (44.9)	529 (41.6)	23 (3.9)	<0.001
20 mg + 10 mg, *n* (%)	571 (36.4)	36 (27.1)	31 (39.7)	474 (37.3)	21 (3.6)	<0.001
40 mg + 10 mg, *n* (%)	73 (4.6)	11 (8.3)	4 (5.1)	55 (4.3)	2 (0.3)	<0.001
**Atorvastatin**, *n* (%)	420 (26.8)	40 (30.1)	24 (30.8)	332 (26.1)	381 (65.9)	<0.001
40 mg, *n* (%)	236 (15.0)	32 (24.1)	16 (20.5)	166 (13.1)	251 (43.4)	<0.001
80 mg, *n* (%)	184 (11.7)	8 (6.0)	8 (10.3)	166 (13.1)	130 (22.5)	<0.001
**Atorvastatin + Ezetimibe**, *n* (%)	331 (21.1)	19 (14.3)	9 (11.5)	293 (23.1)	47 (8.1)	<0.001
40 mg + 10 mg, *n* (%)	136 (8.6)	11 (8.3)	6 (7.7)	111 (8.7)	36 (6.2)	0.065
80 mg + 10 mg, *n* (%)	195 (12.4)	8 (6.0)	3 (3.8)	182 (14.3)	11 (1.9)	<0.001

Valid percentages are used otherwise stated. ^1^ Chi-square.

## Data Availability

Data are contained within the article or supplementary material. The datasets used and/or analyzed during the current study are available from the corresponding author upon reasonable request.

## Supplementary Materials

The following supporting information can be downloaded at: https://www.mdpi.com/article/10.3390/jcm12093187/s1, Figure S1: Flowchart of the patient selection process; Table S1: Changes in LLT due to safety reasons (*n* = 57); Table S2: Clinical characteristics associated with the type of statin therapy (*n* = 1570).
