# Supplementary material for: Real-World Attainment of Low-Density Lipoprotein Cholesterol Goals in Patients at High Risk of Cardiovascular Disease Treated with High-Intensity Statins: The TERESA Study
Source: J Clin Med. 2023 Apr 28;12(9):3187. doi: 10.3390/jcm12093187 (PMC10179558; doi:10.3390/jcm12093187)
Supplement: Supplementary file 1 [file jcm-12-03187-s001.zip › jcm-2329693-supplementary.pdf]

Supplementary Materials

Supplementary Figure S1. Flowchart of the patient selection process

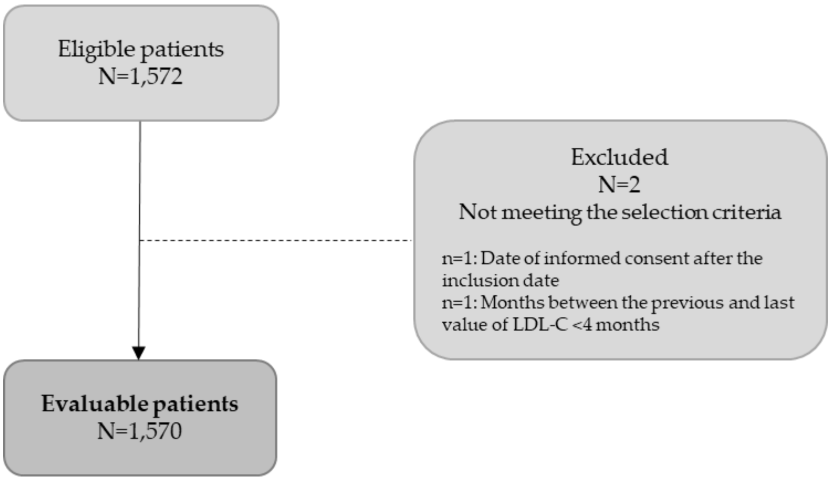

Supplementary Table S1. Changes in LLT due to safety reasons (N=57)

Prior LLT	Current LLT				
	Rosuvastatin	Rosuvastatin + ezetimibe	Atorvastatin	Atorvastatin + ezetimibe	Overall
**Rosuvastatin**	0 (0.0)	3 (5.3)	0 (0.0)	2 (3.5)	5 (8.8)
**Rosuvastatin + ezetimibe**	1 (1.8)	0 (0.0)	0 (0.0)	0 (0.0)	1 (1.8)
**Atorvastatin**	9 (15.8)	30 (52.6)	1 (1.8)	3 (5.3)	43 (75.4)
**Atorvastatin + ezetimibe**	0 (0.0)	5 (8.8)	2 (3.5)	1 (1.8)	8 (14.0)
**Overall**	10 (17.7)	38 (66.7)	3 (5.3)	6 (10.5)	57 (100.0)

LLT, lipid-lowering therapy.

Supplementary Table S2. Clinical characteristics associated with the type of statin therapy (N=1,570)

Variable	Statin therapy		p-value[1]
	Rosuvastatin N (%)	Atorvastatin N (%)	
**Cardiovascular disease**			
Yes	600 (49.3)	616 (50.7)	<0.001
No	219 (61.9)	135 (38.1)	
**Arterial hypertension**			
Yes	604 (52.6)	544 (47.4)	0.569
No	215 (50.9)	207 (49.1)	
**Familial hypercholesterolemia**			
Yes	88 (72.7)	33 (27.3)	<0.001
No	731 (50.4)	718 (49.6)	
**Chronic kidney disease**			
Yes	61 (46.2)	71 (53.8)	0.172
No	758 (52.7)	680 (47.3)	
**Microalbuminuria**			
Yes	95 (58.3)	68 (41.7)	0.115
No	724 (51.5)	683 (48.5)	
**Diabetes mellitus**			
Yes	268 (49.2)	277 (50.8)	0.090
No	551 (53.8)	474 (46.2)	
**Retinopathy**			
Yes	48 (60.8)	31 (39.2)	0.133
No	771 (51.7)	720 (48.3)	
**Neuropathy**			
Yes	23 (51.1)	22 (48.9)	>0.999
No	796 (52.2)	729 (47.8)	

[1]Chi-square
